# Elderly patients have an altered gut-brain axis regardless of the presence of cirrhosis

**DOI:** 10.1038/srep38481

**Published:** 2016-12-06

**Authors:** Jasmohan S. Bajaj, Vishwadeep Ahluwalia, Joel L. Steinberg, Sarah Hobgood, Peter A. Boling, Michael Godschalk, Saima Habib, Melanie B. White, Andrew Fagan, Edith A. Gavis, Dinesh Ganapathy, Phillip B. Hylemon, Karen E. Stewart, Raffi Keradman, Eric J. Liu, Jessica Wang, Patrick M. Gillevet, Masoumeh Sikaroodi, F. Gerard Moeller, James B. Wade

**Affiliations:** 1 Division of Gastroenterology, Hepatology and Nutrition, Virginia Commonwealth University and McGuire VA Medical Center, Richmond, VA, USA; 2 Department of Psychiatry, Virginia Commonwealth University and McGuire VA Medical Center, Richmond, VA, USA; 3 Division of Geriatrics, Virginia Commonwealth University and McGuire VA Medical Center, Richmond, VA, USA; 4 Department of Microbiology and Immunology, Virginia Commonwealth University and McGuire VA Medical Center, Richmond, VA, USA; 5 Microbiome Analysis Center, George Mason University, Manassas, VA, USA

## Abstract

Cognitive difficulties manifested by the growing elderly population with cirrhosis could be amnestic (memory-related) or non-amnestic (memory-unrelated). The underlying neuro-biological and gut-brain changes are unclear in this population. We aimed to define gut-brain axis alterations in elderly cirrhotics compared to non-cirrhotic individuals based on presence of cirrhosis and on neuropsychological performance. Age-matched outpatients with/without cirrhosis underwent cognitive testing (amnestic/non-amnestic domains), quality of life (HRQOL), multi-modal MRI (fMRI go/no-go task, volumetry and MR spectroscopy), blood (inflammatory cytokines) and stool collection (for microbiota). Groups were studied based on cirrhosis/not and also based on neuropsychological performance (amnestic-type, amnestic/non-amnestic-type and unimpaired). Cirrhotics were impaired on non-amnestic and selected amnestic tests, HRQOL and systemic inflammation compared to non-cirrhotics. Cirrhotics demonstrated significant changes on MR spectroscopy but not on fMRI or volumetry. Correlation networks showed that *Lactobacillales* members were positively while *Enterobacteriaceae* and *Porphyromonadaceae* were negatively linked with cognition. Using the neuropsychological classification amnestic/non-amnestic-type individuals were majority cirrhosis and had worse HRQOL, higher inflammation and decreased autochthonous taxa relative abundance compared to the rest. This classification also predicted fMRI, MR spectroscopy and volumetry changes between groups. We conclude that gut-brain axis alterations may be associated with the type of neurobehavioral decline or *inflamm-aging* in elderly cirrhotic subjects.

The rapidly aging population, with the accompanying neuro-cognitive sequelae such as dementia, is a major medical and psychosocial burden^1^. This maturing of the population has also affected patients with cirrhosis, who, in addition to the cognitive issues related to aging, are also prone to hepatic encephalopathy (HE)^2^. Elderly subjects can have issues with amnestic (memory-related) and non-amnestic (unrelated to memory) cognitive dysfunction, which may be reflected in geriatric cirrhotic patients as well. The subclinical phase, known as covert HE (CHE) usually affects non-amnestic domains such as attention, visuo-motor coordination and executive function^3^. This has been shown in prior studies using mixed tests of memory and attention in which younger cirrhotics are more likely to be impaired on attention and visuo-spatial domains rather than amnestic domains such as delayed memory^4^. Further characterization of the interaction between dementia (mostly associated with amnestic issues) and HE in elderly cirrhotics is critical because an increasing number of these patients are being evaluated for liver transplant^5^. Patients with HE, unlike those with dementia, can expect a reasonable improvement in cognition after transplant and are considered appropriate for listing^6^. The initial differentiation between these groups of patients is often performed at the level of neuro-psychological evaluation but the utility of this in elderly cirrhotics is unclear.

There is also emerging evidence regarding the impaired gut-brain axis in the setting of systemic inflammation in younger cirrhotics and in non-cirrhotic patients with amnestic impairment^7^,^8^,^9^,^10^,^11^. The concept of “*inflamm-aging*” has been studied in elderly patients with a strong focus on an altered gut-brain axis^12^. However these inflammatory and gut microbial changes need to be studied in the context of elderly cirrhotic patients.

We hypothesized that elderly cirrhotic patients with concomitant amnestic and non-amnestic cognitive impairment, will have poor HRQOL, higher systemic inflammation, and decreased relative abundance of beneficial gut microbiota, associated with changes in multi-modal brain MRI compared to unimpaired subjects.

## Results

93 potential patients were referred by the geriatric and gastroenterology clinics for possible inclusion from January 2015 to March 2016. Eight were not able to complete the MMSE and had diagnosed dementia. Six patients had co-morbid conditions that precluded participation, and three were on psycho-active medications. Ultimately 76 patients were included, 39 of whom were cirrhotic and 37 were non-cirrhotic. Based on the cognitive algorithm the neuropsychologist divided patients into unimpaired (n = 23), amnestic-type (n = 25), and amnestic/non-amnestic type (n = 28). The kappa of this classification between the two psychologists was 0.89. While the neuropsychologist classified one patient as amnestic/non-amnestic type, the psychologist considered him amnestic type, while another patient classified as amnestic type was considered unimpaired.

### Cognitive performance and HRQOL

Cirrhotic and non-cirrhotic patients had similar demographics and MMSE (Table 1). Cirrhotics demonstrated worse overall RBANS performance, but not delayed memory or language subtests. Cirrhotics performed worse on PHES and EncephalApp compared to non-cirrhotics. Portions of the other amnestic tests (HVLT total recall and Similarities test) were also impaired in cirrhotic patients, who also had a worse HRQOL on both SIP and PROMIS (Table 1).

The groups divided according to neuropsychological performance had corresponding cognitive impairments as expected without differences in demographics (Table 2). The amnestic/non-amnestic group had the highest proportion of cirrhotic patients and had the highest HRQOL impairment. In the cirrhosis group, there were no statistically significant correlations between MELD score and cognitive performance or HRQOL.

### Multi-modal MRI analysis

#### fMRI

**Figure 1:**
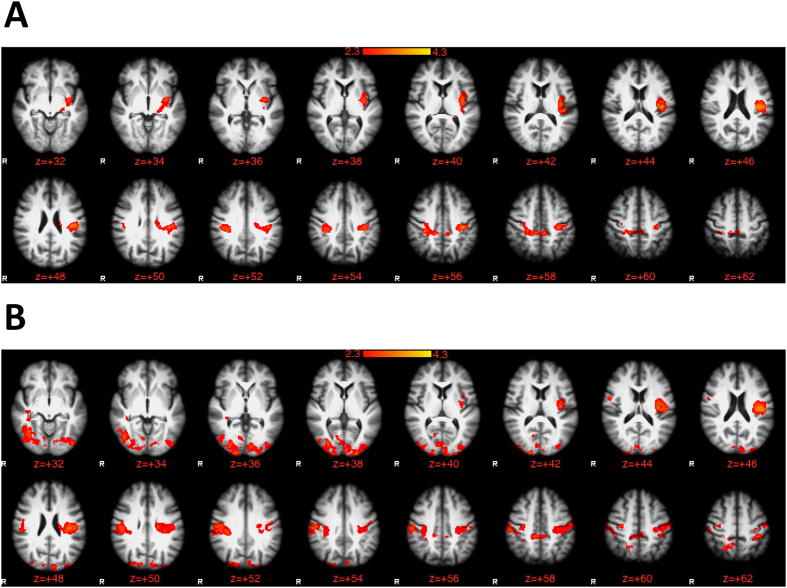
Group difference contrasts on brain activation on functional MRI during correct inhibition to lures on the inhibitory control tests using a mixed-effects analysis. The cluster-forming threshold was created with z = 2.3 with a corrected p < 0.05. The red-yellow schema is based on levels of gradation of differences from 2.3 to >4 between groups. (**A**) Comparison of amnestic/non-amnestic type to amnestic type patients in which amnestic/non-amnestic type patients required a greater brain activation extent to achieve the similar response on lure inhibition compared to amnestic type patients. (**B**) Comparison of amnestic/non-amnestic type to unimpaired patients in which amnestic/non-amnestic type patients required a greater brain activation extent to achieve the similar response on lure inhibition compared to unimpaired subjects.

When cirrhotics were compared to non-cirrhotic patients, there were no significant differences in brain activation extent. However, based on cognitive divisions, correct inhibition revealed widespread activation in areas within the dorsolateral prefrontal, paracingulate, posterior cingulate, precuneous, supplementary motor area and posterior parietal cortices in all groups. Amnestic/non-amnestic patients had significantly higher activation during inhibition compared to the other groups in several regions including central opercular cortex, postcentral gyrus and superior parietal lobule. No differences were found between amnestic and unimpaired subjects (Table 3, Fig. 1A,B).

#### MR Spectroscopy

Cirrhotic subjects had lower creatine ratios of myoinositoI and NAA + NAAG, and higher creatine ratio of Glutamate + Glutamine in the anterior cingulate cortex. Using the neuropsychological classification, both amnestic/non-amnestic patients had a lower mI/creatine ratio and a lower NAA + NAAG creatine ratio than amnestic patients (Table 4). Amnestic/non-amnestic patients also had a lower NAA + NAAG creatine ratio than unimpaired subjects (p = 0.005).

#### Volumetric analysis

Total brain volumes were similar between cirrhotic compared to non-cirrhotic subjects. When divided based on neuropsychology, the amnestic-type patients had lower white matter, gray matter and total brain volume as well as Hippocampal and left thalamic volume compared to the other two groups (Table 5). Right thalamic volumes in amnestic group were only lower than the amnestic/non-amnestic group.

### Systemic inflammatory cytokines

Serum IL-6 was higher in cirrhotic compared to non-cirrhotic subjects (median 2.6 vs 1.0 pg/ml, p = 0.002), as was serum endotoxin (median 0.50 vs. 0.34 Eu/ml, p = 0.05). On the other hand serum IFN-γ (0.55 vs 1.10 pg/ml, p = 0.48) and PGE2 (146.7 vs. 125.2 pg/ml, p = 0.73) were similar. Using the neuropsychological performance, a progressive significant increase in IFN-γ (0.0 vs 1.1 vs 1.4 pg/ml, p = 0.007) was seen. IL-6 levels were only high in patients with amnestic/non-amnestic impairment (1.0 vs. 1.0 vs. 2.6 pg/ml, p = 0.02). There was no significant difference in PGE2 levels (140.0 vs. 121.3 vs 101.6, p = 0.89) or serum endotoxin between the groups based on neuropsychological performance (0.36 vs. 0.38 vs. 0.45 Eu/ml, p = 0.64).

### Gut microbiota changes and correlation networks

**Figure 2:**
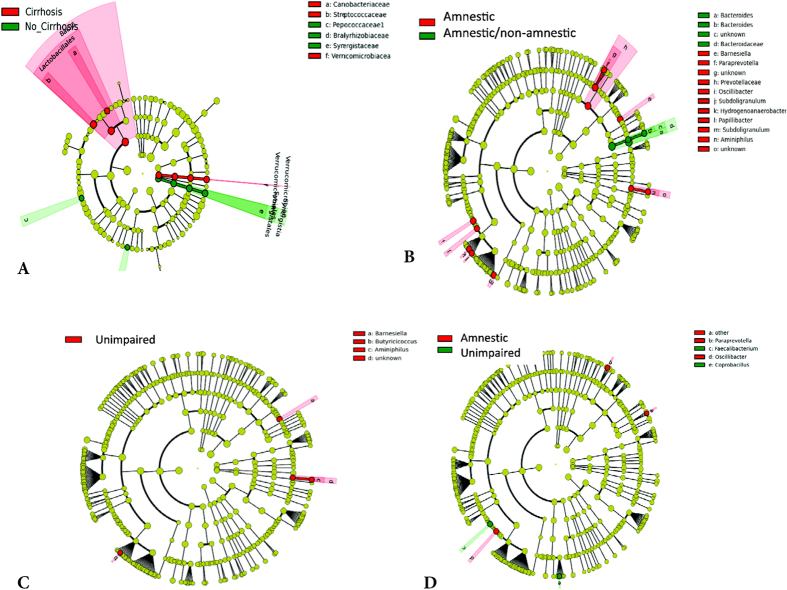
Microbiota changes. The linear discriminant analysis effect size (LEFSe) cladogram shows differences in bacterial taxa between groups. The concentric circles indicate phylogenetic taxa from phylum (innermost) to family/genus. Taxa different between compared groups are coded in red or green as indicated in (**A–D**). (**A**) LEfSe comparison between cirrhotic patients and non-cirrhotic patients. The cladogram shows the phylogenetic relationship between the bacterial families that were higher in non-cirrhotic (green) compared to cirrhotic patients that are represented in the red. (**B**) LEFse comparison between amnestic/non-amnestic type and amnestic type patients. The cladogram shows the phylogenetic relationship between the bacterial taxa that were higher in amnestic/non-amnestic type (green) compared to amnestic type patients that are represented in the red. (**C**) LEfSe comparison between unimpaired and amnestic/non-amnestic type patients. The cladogram shows the phylogenetic relationship between the bacterial taxa that were higher in unimpaired patients (red) compared to amnestic/non-amnestic patients. (**D**) LEfSe comparison between unimpaired and amnestic patients. The cladogram shows the phylogenetic relationship between the bacterial taxa that were higher in unimpaired patients (green) compared to amnestic patients (red).

Cirrhotic subjects showed a higher relative abundance of members of *Lactobacillales (Carnobacteriaceae*, *Streptococcaceae* and *Verrucomicrobiae*), and a lower relative abundance of *Synergisticeae* and *Peptococcaceae* (Fig. 2A). Using neuropsychological divisions, amnestic/non-amnestic-type patients compared to amnestic-type had a significantly lower relative abundance of genera belonging to autochthonous (*Subdoligranulum, Oscillibacter*) and oral-origin families (*Porphyromonadaceae* and *Prevotellaceae*), and a higher *Bacteroides* abundance. These genera were also present in amnestic-type patients compared to unimpaired; however unimpaired subjects had a higher relative abundance of *Fecalibacterium*. A higher relative abundance of a butyrate-producing genus (*Butyricicoccus*) was found in unimpaired compared to amnestic/non-amnestic -type subjects (Fig. 2B–D).

#### Correlation networks

**Figure 3:**
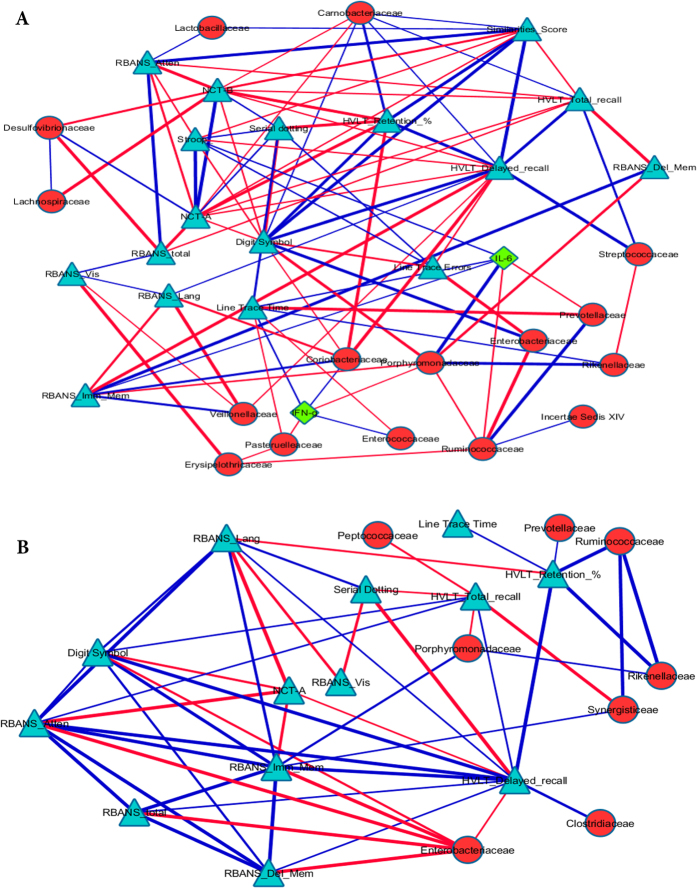
Correlation network analysis. Correlation networks were visualized in Cytoscape. The nodes are microbiota (red circles), cognitive tests (blue triangles) and inflammatory cytokines (green diamonds). Edges joining the nodes are color and size-coded. Blue edges indicate a positive (r > 0.6) and red lines indicate a negative (r < −0.6) correlations while thicker lines indicate a higher significance beyond p < 0.01. RBANS: Repeatable battery for assessment of neuropsychological status, Imm_Mem: immediate memory domain, Del_Mem: delayed memory domain, Att: Attention domain, Vis: Visuo-spatial domain, Lang: Language domain, HVLT: Hopkins Verbal Learning test, Retention %: retention index, NCT-A: number connection test −A, NCT-B: number connection test-B, Line trace time: Line tracing test time, Stroop: EncephalApp Stroop total OffTime + OnTime. A low score on serial dotting, NCT-A, NCT-B, Line trace time and Stroop indicate good performance while a high score on the remaining cognitive tests indicate a good performance. (**A**) Correlation network for cirrhotic elderly subjects. Significant positive correlations were found between autochthonous bacteria (*Ruminococcaceae*, *Lachnospiraceae*) and good cognition and lower systemic inflammation while the reverse was seen for *Enterobacteriaceae* and *Porphyromonadaceae. Lactobacillaceae*, *Carnobacteriaceae* and *Streptococcaceae*, all of which belong to *Lactobacillales* and are higher in cirrhotic compared to non-cirrhotic individuals, were associated with good cognitive performance. As expected most cognitive tests were linked with each other in the expected directions. (**B**) Correlation network for non-cirrhotic elderly subjects. Significant positive correlations were seen between *Ruminococcaceae* and good cognition and negative ones between *Enterobacteriaceae* and cognitive performance. *Peptococcaceae* and *Synergistaceae*, which were higher in non-cirrhotics compared to cirrhotics, were actually associated with a poor cognitive performance. The correlations of *Porphyromonadaceae* were largely associated with poor cognitive performance.

In the cirrhosis group, members of *Lactobacillales (Streptoccaceae*, *Carnobacteriaceae* and *Lactobacillaceae*) were linked with good cognition on memory-based tests, while beneficial autochthonous taxa such as *Ruminococcaceae* and *Lachnospiraceae* were linked with memory-unrelated test performance. In contrast potentially pathogenic taxa such as *Enterobacteriaceae* were negatively linked with memory-unrelated performance (Fig. 3A,B). In the non-cirrhosis group, the linkage pattern between cognition, autochthonous and potentially pathogenic taxa was similar to cirrhotic group. In addition, LEfSe-discriminated taxa associated with the non-cirrhosis group in comparison with cirrhotics, *Synergisticaeae and Peptococcaceae*, were associated with poor cognitive performance (negatively associated with HVLT score).

## Discussion

This study demonstrates that elderly cirrhotic patients have a significantly impaired cognitive performance and HRQOL, associated with systemic inflammation, gut dysbiosis and altered MR spectroscopic findings compared to age-matched non-cirrhotic subjects. Furthermore, this altered gut-liver-brain axis, especially from a multi-modal MRI perspective, is refined when neuropsychological profile rather than cirrhosis itself is used as a classifier. Changes in systemic inflammation and gut microbiota that track the neuropsychological classification suggest a potential biological basis for this division that transcends the diagnosis of cirrhosis. Similarly, neuropsychological impairment was found to be the earliest biomarker for predicting progression of mild cognitive impairment and Alzheimer’s disease^13^,^14^.

The growing elderly cirrhotic population, who could have concomitant amnestic and non-amnestic cognitive deficits, remains an important concern for clinicians and neuropsychologists. The converging effects of CHE and age-related changes suggestive of mild cognitive impairment in these patients could impact the HRQOL and potentially disease progression. Our findings demonstrated that the neurocognitive profile of elderly cirrhotic patients was largely similar to what could be expected in younger cirrhotics compared to non-cirrhotic individuals with a few exceptions^3^. Elderly cirrhotic subjects had impairment on selected memory-related tests that would not be expected in younger cirrhotic patients^3^. The relationship between cirrhosis and aging was therefore further explored using the neuropsychological classification. This classification showed that almost half of the patients classified as amnestic type and more than three quarters of amnestic/non-amnestic type had underlying cirrhosis. This higher burden of cognitive impairment in cirrhotic patients, regardless of classification modality, translated into a worse HRQOL that spanned most areas of daily functioning. Therefore elderly cirrhotic patients, even without prior overt HE, are likely to have greater amnestic impairment, which translates into a poor HRQOL compared to non-cirrhotic individuals.

While the relatively simple classification according to underlying cirrhosis was important to define HRQOL changes, it did not readily translate into corresponding changes in fMRI or volumetric analyses. On the other hand the neuropsychological classification determined that patients with amnestic/non-amnestic impairment required greater neuronal recruitment of the visuo-spatial network to achieve the same response compared those with amnestic impairment alone, or unimpaired subjects regardless of cirrhosis^15^. A similar pattern was observed on volumetry where cirrhosis vs. no-cirrhosis did not significantly differ while the neuropsychological classification clearly separated the groups. Interestingly, hippocampal and brain volume decrease was found in those with predominant amnestic type. A lower brain volume has been associated with non-cirrhotic amnestic impairment and our study found similar trends in patients with amnestic impairment, which included cirrhotic patients^13^. The relative higher brain volumes in the predominantly cirrhotic amnestic/non-amnestic group likely reflect cirrhosis-associated brain edema^16^. These findings were further extended by the MR spectroscopy data. This showed that elderly cirrhotic patients, in addition to demonstrating ammonia-associated changes of higher creatine-adjusted values of Glx and lower mI, and had a lower creatine-adjusted NAA + NAAG, which is a neuronal marker described again in non-cirrhotic amnestic impairment^17^. This MR spectroscopic classification was further refined by the cognitive classification that reaffirmed the lower creatine ratios of NAA + NAAG and mI in the most impaired groups. Therefore, from a multi-modal MRI perspective, where changes are often subtle and may be subclinical, a division based on neuropsychological performance may help define brain dysfunction better than a simplistic cirrhosis/no-cirrhosis classification.

However, the pathophysiology of this cognitive impairment could span several other organ systems, given the complex interaction between systemic inflammation, gut microbial dysbiosis and brain function in younger cirrhotic patients^8^,^18^,^19^. When taken as a whole group, patients with cirrhosis regardless of neuropsychological performance had higher relative abundances of *Lactobacillales* members. These taxa were positively linked with good performance on memory-based tests, which could potentially explain the lack of major differences on these tests between the cirrhotic and non-cirrhotic groups. However, the non-cirrhotic group had higher relative abundances of *Synergistaceae* and *Peptococcaceae*, which were related to poor amnestic performance. Prior known potentially beneficial, autochthonous families such as *Ruminococcaceae* and *Lachnospiraceae* were positively linked, while potentially pathogenic taxa such as *Enterobacteriaceae* were negatively linked to good cognition regardless of cirrhosis^19^.

Intriguingly changes in gut microbiota persisted when groups were classified neuropsychologically. *Fecalibacterium* and *Butyricoccus*, which are potentially probiotic genera associated with inflammation suppression and butyrate production respectively, were higher in cognitively unimpaired subjects^20^,^21^,^22^. On the other hand, genera belonging to beneficial, autochthonous families were less likely in patients with amnestic/non-amnestic type compared to those with amnestic type alone^19^. Interestingly, *Porphromomadaceae*, has been associated with neuronal dysfunction in younger cirrhotics, were more likely to be present in amnestic patients and was associated with poor cognition in the cirrhosis correlation network^9^,^11^, alluding to the predominant neuronal role in the progression to dementia. Interestingly, unlike in younger cirrhosis vs. no-cirrhosis comparisons, we did not find members of *Proteobacteria* phylum as differentiators. This could reflect prior studies that show that elderly subjects have a baseline higher *Proteobacteria* abundance overall, and could also explain the relatively insignificant overall changes in endotoxin^23^. The other inflammatory markers were chosen to represent innate immunity (IFN-γ), prostaglandin pathway (PGE2) and systemic inflammation (IL-6). PGE2 was analyzed because prior non-cirrhotic human and animal reports have alluded to it as a marker of pre-clinical dementia, but it was not able to differentiate the current population^24^. A higher IL-6 is often seen in cirrhotic patients, which could explain the higher values in the amnestic/non-amnestic group^25^. A higher IFN-γ in both cognitively impaired groups could reflect a non-specific innate response to the bacterial dysbiosis^26^.

This differential systemic milieu could link microbial and brain changes but endotoxemia does not seem to play an important role^27^. However, further mechanistic studies are needed. Beneficial changes in butyrate and inflammation have been shown with use of synbiotics in elderly patients, noting that these factors may be another aspect to improve the impaired inflammatory-immune basis of *inflamm-aging*^8^,^12^,^28^. Further studies focusing on improving the altered gut-brain axis in cirrhotic elderly patients and potentially using specific stool bacteria as biomarkers for disease progression are needed.

We excluded OHE patients, who by definition have worse cognition, brain MRI results and gut dysbiosis compared to others, because it could have influenced the interpretation of cognitive tests, and gut microbiota analysis^19^,^27^. However, we were still able to find differences in the population with subclinical cognitive dysfunction, and would expect these changes to be magnified in future studies with elderly OHE patients. Another limitation of this study is that it represents a highly selected subject sample without significant co-morbid conditions. The relative contribution of cirrhosis, dysbiosis and aging on the ultimate cognitive and brain MR function is difficult to delineate given the sample size, and larger studies are required.

Our results suggest that an altered gut-brain axis occurs in elderly patients with differential changes based on the underlying cirrhosis and the type of cognitive dysfunction. Elderly cirrhotic patients are likely to have significant amnestic or memory-related impairment, which is usually associated with dementia and translates into a poor HRQOL. A classification based on neuropsychological performance further refines changes in multi-modal MRI markers of functional and structural cortical connectivity. Further studies are needed in more advanced patients to define the effect of these changes on outcomes, and to utilize the gut microbiota as an alternative approach to diagnose and treat cognitive disorders in the growing elderly cirrhotic population.

## Methods

We enrolled consecutive outpatients between the 65 and 85 years of age after informed consent. We excluded patients with significant physical (congestive heart failure, chronic obstructive pulmonary disease, HIV, malignancies, dialysis-dependent, diabetes with HbA1c > 7%) and neuropsychiatric conditions (established dementia, prior stroke, bipolar disorder, schizophrenia, Parkinson’s disease, multiple sclerosis, seizures). We also excluded all on anti-psychotic, anti-seizure, benzodiazepines and chronic anti-depressants other than selective serotonin reuptake inhibitors (SSRI). We did not exclude patients on chronic SSRI given the drugs’ minimal effect on cognitive performance in cirrhosis^29^. We also excluded subjects with alcohol misuse or illicit drug use within 3 months, and those who had used laxatives, probiotics and antibiotics within the last six weeks. Cirrhotic patients were excluded if they had current/prior overt hepatic encephalopathy (OHE)^19^,^27^. After enrollment, all subjects underwent mini-mental status testing (MMSE) and the Beck Depression Inventory-II (BDI-II). A score <25 on MMSE and >12 on BDI-II excluded them from further study^30^. Demographic information and severity of cirrhosis were recorded. Patients then underwent the following (1) Cognitive testing (2) HRQOL assessment (3) Stool/Blood Sample Collection (4) Multi-modal brain MRI and (5) Psychologist interview.

### Cognitive Battery

These batteries characterize (a) predominantly non-amnestic impairment [Psychometric hepatic encephalopathy score (PHES), and EncephalApp Stroop]^3^,^31^, (b) predominantly amnestic impairment [Hopkins Verbal Learning test (HVLT)^32^] and (c) batteries that evaluate both amnestic and non-amnestic impairment [Repeatable Battery for the Assessment of Neuropsychological Status (RBANS)^33^ and Similarities subtests^34^]. The RBANS^33^ assesses 5 cognitive domains (immediate and delayed memory, visuo-spatial skills, attention, and language). While it was originally developed for screening of dementia, it has also been used in HE, with a predominant “subcortical” pattern of dysfunction^33^,^4^.

HRQOL assessment was performed using the Sickness Impact Profile (SIP) and the computerized adaptive PROMIS tools^35^. We collected serum for inflammatory cytokines, and stool for microbiota analysis using published methods^36^.

Inflammatory cytokines analyzed were IL-6, Interferon-γ(IFN-γ) and prostaglandin E2(PGE2)^24^ using ELISA and endotoxin was assessed using LAL assay (Assaygate, Ijamsville, MD)^19^. Stool microbiota composition was determined using published multi-tagged sequencing techniques^37^ and analyzed using LEFSe (Linear Discriminant Analysis Effect Size) between the study groups^38^. Correlation network analysis between cognitive testing results, microbiota and inflammatory cytokines was performed in the cirrhotic group and in the non-cirrhotic group using a customized R package^11^. Only correlations that were p < 0.01 and r > 0.6 or <−0.6 were studied.

Patients then underwent multi-modal brain MRI on the same day as these procedures. The MRI consisted of (1) fMRI, (2) MR spectroscopy, and (3) volumetric analysis

#### fMRI ICT task

During this task the subject was asked to respond to targets and withhold responses to lures, on inhibitory control test, a validated go/no-go task, while in the scanner^39^. A brief training session was performed prior to the MRI scan. Each subject underwent six runs of the ICT inside the scanner (supplementary information).

#### fMRI data analysis

fMRI data analysis was carried out using the FMRI Expert Analysis Tool v 5.98 part of FSL (FMRIB’s Software Library)^40^. After standard preprocessing, a time-series statistical analysis was carried out with local autocorrelation correction using the General Linear Model. The model included correct response to target and correct inhibition to lures as contrast of interest. Incorrect response to lure, random responses and six motion parameters were added as contrasts of no interest. Group average statistical maps were created in Montreal Neurological Institute (MNI) standard space for all correct inhibition to lures trials. The MNI template is an internationally recognize standard for anatomical localization of brain activation observed on fMRI. Also, statistical maps to evaluate group differences were generated and thresholded using cluster-based thresholding.

#### Spectroscopy analysis

The choline, creatine, myo-inositol, N-acetylaspartate + N-acetylaspartate glutamate (NAAA + NAAG) and glutamate + glutamine (Glx) complex peak areas were computed using a quantitative assessment of the metabolite concentration by means of LCModel software with the creatine concentration ratio^41^. These metabolites were chosen based on prior HE research^16^.

#### Volumetric analysis

Brain tissue volume, normalized for subject head size, was estimated with SIENAX^42^. Additionally, we also estimated hippocampal and thalamus volumes using part of FSL^43^ and normalized them for head size.

##### Study Approval and Consents

All participants gave informed consent. The study is approved by the Institutional Review Boards at Virginia Commonwealth University and McGuire VA Medical Center and all experiments were performed in accordance with relevant guidelines and regulations.

Analysis of the subjects was performed using two major classifications:

1. Cirrhotic vs. non-cirrhotic patients and
2. Subjects without impairment vs. those with predominant amnestic impairment vs. those with both amnestic and non-amnestic impairment regardless of cirrhosis.

In arriving at the classification the neuropsychologist reviewed, and applied age correction, to the subject’s raw cognitive scores and conducted a standardized interview with each subject and their companion, if available. During the interview academic, vocational, and substance abuse history was obtained. Subjects whose cognitive performance on all the measures ranged from –2 standard deviations and above were considered unimpaired. Subjects with focal impairment ≥2 SD on ≥2 learning/memory subtests were classified as amnestic type. Finally, subjects were considered amnestic/non-amnestic type if they were impaired ≥2 SD on measures in >2 domains, such as information/psychomotor speed and learning/memory. The interview notes and cognitive performance were then reviewed by another psychologist (KS) who was blinded to the original division by the neuropsychologist. A Kappa score between the divisions was calculated for concordance.

#### Statistical analysis

All statistical analyses were performed using the SPSS (version 12, SPSS Inc., Chicago, IL, USA) with appropriate tests. Laboratory tests, neuropsychological tests, HRQOL tests, volumetrics and MRS were compared between groups by the two-tailed unpaired t-test or ANOVA based on the divisions. Multiple comparison correction was done wherever applicable using Holm-Bonferroni method. Specialized analyses (microbiota, fMRI, correlation networks are mentioned in individual sections). A sample size of at least 16 in each group was deemed to be appropriate with an 80% power based on our prior studies on gut-brain analysis in younger patients with cirrhosis compared to healthy controls^9^.

## Additional Information

**How to cite this article**: Bajaj, J. S. *et al*. Elderly patients have an altered gut-brain axis regardless of the presence of cirrhosis. *Sci. Rep.* **6**, 38481; doi: 10.1038/srep38481 (2016).

**Publisher's note:** Springer Nature remains neutral with regard to jurisdictional claims in published maps and institutional affiliations.

## Supplementary Material

Supplementary Information

## Figures and Tables

**Table 1:** Clinical Comparison between Cirrhotic and Non-Cirrhotic Subjects.

	No Cirrhosis (n = 37)	Cirrhosis (n = 39)	P-value
Age (years)	73.9 ± 5.9	72.9 ± 5.4	0.43
Gender (% men)	68%	77%	0.36
Education (years)	15.2 ± 3.6	14.9 ± 2.7	0.11
Diabetes (%)	12 (32%)	16 (41%)	0.58
Alcohol abuse history (%)	6 (16%)	13 (33%)	0.09
Hypertension (%)	15 (41%)	12 (31%)	0.33
Depression on SSRI (%)	9 (24%)	7 (18%)	0.50
Hyperlipidemia (%)	16 (43%)	12 (31%)	0.26
Mini-mental status	28.8 ± 1.4	28.4 ± 1.4	0.22
HVLT			
HVLT Total recall	22.7 ± 5.6	19.1 ± 5.8	0.04
HVLT delayed recall	7.1 ± 2.9	6.9 ± 3.1	0.79
HVLT retention %	74.1 ± 22.5	82.7 ± 33.3	0.21
Similarities test	27.7 ± 4.6	24.3 ± 6.3	0.01
RBANS			
Total score	103.4 ± 18.7	91.3 ± 15.5	0.004
Visuospatial	109.7 ± 17.5	94.4 ± 19.8	0.001
Immediate Memory	101.7 ± 16.6	90.6 ± 17.6	0.04
Language	98.2 ± 13.5	95.3 ± 8.5	0.29
Attention	106.3 ± 17.7	96.1 ± 17.4	0.02
Delayed Memory	98.0 ± 18.8	92.1 ± 16.9	0.09
EncephalApp Stroop			
Stroop Off Time (sec)	86.1 ± 27.7	100.5 ± 28.4	0.05
On Time (sec)	105.6 ± 44.3	127.8 ± 43.6	0.06
Median runs On	5.0	6.0	0.02
Median runs Off	6.0	6.0	0.47
OffTime + OnTime (sec)	182.6 ± 68.7	214.5 ± 66.5	0.05
PHES			
Number connection-A (sec)	45.0 ± 29.4	52.7 ± 28.3	0.25
Number connection-B (sec)	114.8 ± 81.9	145.0 ± 102.0	0.15
Digit Symbol (raw score)	54.3 ± 19.1	45.2 ± 17.9	0.04
Line Tracing test (sec)	106.4 ± 59.1	114.3 ± 59.5	0.56
Line Tracing errors	30.9 ± 31.9	41.4 ± 31.4	0.16
Serial dotting (sec)	57.2 ± 21.2	82.9 ± 38.1	<0.001
Median PHES	−1	−7	0.01
HRQOL assessments			
SIP			
Total score	3.1 ± 5.8	10.8 ± 11.4	<0.0001
Psychosocial domain	2.6 ± 4.6	9.7 ± 12.8	0.001
Physical domain	2.6 ± 5.5	10.4 ± 12.0	<0.0001
Median Age-adjusted PROMIS scores			
Anger	39.5	58.0	0.02
Anxiety	66.0	63.0	0.63
Depression	45.5	49.0	0.25
Fatigue	35.0	54.0	0.05
Physical function*	79.0	48.0	0.004
Social activity*	76.5	47.0	0.003
Social Role*	69.0	32.0	<0.0001
Sleep disturbance	44.5	70.0	0.02
Wake disturbances	29.5	58.0	0.04

P value using ANOVA or Chi-square test as appropriate, ^*^a low score on these PROMIS variables indicates worse function, while a low score on the others indicates good function. PHES: psychometric hepatic encephalopathy score, PROMIS: patient-reported outcome measurement information system, SIP: Sickness Impact Profile, HRQOL: Health-related quality of life, RBANS: repeatable battery for assessment of neuropsychological status, HVLT: Hopkins Verbal Learning Test.

**Table 2:** Demographic, cognitive and quality of life variables according to neuropsychological division.

	Unimpaired (n = 23)	Amnestic type (n = 25)	Amnestic/non-amnestic type (n = 28)	P value
Age (years)	73.3 ± 4.4	71.9 ± 5.4	72.7 ± 5.9	0.77
Gender (% men)	55%	64%	60%	0.67
Education (years)	15.8 ± 2.7	16.8 ± 2.4	15.5 ± 2.9	0.12
Cirrhosis (%)	5 (22%)	12 (48%)	22 (78%)†,‡	<0.0001
Diabetes (%)	10 (43%)	7 (28%)	11 (39%)	0.51
Alcohol abuse history (%)	3 (13%)	6 (21%)	10 (36%)	0.18
Hypertension (%)	8 (35%)	11 (44%)	10 (36%)	0.76
Depression on SSRI (%)	4 (17%)	6 (24%)	6 (21%)	0.85
Hyperlipidemia (%)	7 (30%)	13 (52%)	8 (28%)	0.16
Mini-mental status	29 ± 1	28 ± 2	28 ± 1	0.02
HVLT				
HVLT Total recall	25 ± 5	18 ± 4	19 ± 6‡	0.001
HVLT delayed recall	9 ± 2	3 ± 2	7 ± 2†	<0.0001
HVLT retention %	89 ± 18	45 ± 29	86 ± 27†	<0.0001
Similarities test	29 ± 4	26 ± 6	23 ± 6†,‡	0.02
RBANS				
Total score	114.3 ± 13.0	94.1 ± 13.3	88.4 ± 17.6‡	<0.0001
Visuospatial	117.9 ± 14.0	102.1 ± 18.6	95.6 ± 14.9†,‡	<0.0001
Immediate Memory	103.7 ± 14.7	89.7 ± 12.1	86.5 ± 19.3‡	0.004
Language	103.3 ± 10.8	95.1 ± 7.3	92.5 ± 14.0‡	0.01
Attention	110.4 ± 12.9	109.1 ± 15.8	93.1 ± 15.4†,‡	0.001
Delayed Memory	109.3 ± 9.2	82.9 ± 18.4	88.4 ± 20.5‡	<0.001
EncephalApp Stroop				
Stroop Off Time (sec)	82.0 ± 16.2	92.5 ± 26.1	109.4 ± 37.4†,‡	0.012
On Time (sec)	104.0 ± 38.9	114.0 ± 38.9	139.2 ± 58.9†,‡	0.05
Median runs On	5.0	5.5	6.0	0.31
Median runs Off	6.0	6.0	6.5	0.38
OffTime + OnTime (sec)	180.4 ± 36.3	184.4 ± 51.0	248.6 ± 90.4†,‡	0.003
PHES				
Number connection-A (sec)	34.7 ± 10.9	44.6 ± 22.6	65.3 ± 42.5†,‡	0.006
Number connection-B (sec)	86.2 ± 33.4	111.0 ± 67.8	179.5 ± 115.9†,‡	0.002
Digit Symbol (raw score)	57.2 ± 14.9	48.9 ± 16.1	41.9 ± 21.7†,‡	0.03
Line Tracing test (sec)	104.8 ± 35.0	95.6 ± 42.3	121.3 ± 65.5	0.32
Line Tracing errors	25.3 ± 17.7	44.6 ± 42.4	39.5 ± 31.9	0.16
Serial dotting (sec)	54.5 ± 15.7	63.6 ± 32.9	86.6 ± 43.8†,‡	0.01
Median PHES	0	−3	−6†,‡	0.001
HRQOL assessments				
SIP				
Total score	1.4 ± 1.9	6.3 ± 7.5	11.1 ± 13.3†,‡	0.006
Psychosocial domain	1.4 ± 1.8	5.5 ± 5.8	9.0 ± 13.6†,‡	0.03
Physical domain	1.2 ± 2.5	5.5 ± 7.3	11.2 ± 13.5†,‡	0.005
Median Age-adjusted PROMIS scores				
Anger	33.0	48.5	75.5†,‡	0.002
Anxiety	45.0	55.0	87.0†,‡	0.001
Depression	38.0	49.0	64.0†,‡	0.003
Fatigue	26.0	41.5	92.0†,‡	0.006
Physical function*	79.0	62.0	39.5†,‡	0.005
Social activity*	79.0	56.5	33.0†,‡	0.004
Social Role*	62.5	53.5	8.0†,‡	0.008
Sleep disturbance	33.0	37.5	73.0†,‡	0.005
Wake disturbances	26.0	62.5	85.0†,‡	0.003

P value using ANOVA or Chi-square test as appropriate, ^*^a low score on these PROMIS variables indicates worse function, while a low score on the others indicates good function. ^†^p < 0.05 between amnestic/non-amnestic and amnestic type, ^‡^p < 0.05 between amnestic/non-amnestic and unimpaired. PHES: psychometric hepatic encephalopathy score, PROMIS: patient-reported outcome measurement information system, SIP: Sickness Impact Profile, HRQOL: Health-related quality of life, RBANS: repeatable battery for assessment of neuropsychological status, HVLT: Hopkins Verbal Learning Test.

**Table 3:** Activation table of brain areas showing group differences during correct inhibition to lures; no differences were found between cirrhosis vs. no-cirrhosis and between amnestic group and unimpaired patients.

	Cluster Index	Z-score	MNI (mm)		
			X	y	z
**Amnestic/Non-amnestic >Unimpaired**^a^					
*Cluster 2: 2252 voxels*					
Central Opercular Cortex, L	2	3.50	−42	−22	20
Postcentral Gyrus, L	2	3.20	−38	−24	40
Insular Cortex, L	2	3.12	−36	−10	8
*Cluster 1: 1199 voxels*					
Postcentral Gyrus, R	1	3.16	34	−32	40
Posterior Cingulate Gyrus, R	1	3.15	14	−36	42
Superior Parietal Lobule, R	1	2.62	32	−40	58
**Amnestic/Non-amnestic >Unimpaired**^b^					
*Cluster 4: 5395 voxels*					
Temporal Occipital Fusiform Cortex, R	4	3.63	38	−60	−10
Occipital Pole, L	4	3.54	−22	−102	4
Occipital Pole, R	4	3.40	32	−90	30
*Cluster 3: 2670 voxels*					
Central Opercular Cortex, L	3	3.70	−40	−20	22
Insular Cortex, L	3	3.57	−38	−12	14
Superior Parietal Lobule, L	3	3.16	−36	−42	58
Postcentral Gyrus, L	3	3.02	−58	−14	46
*Cluster 2: 1585 voxels*					
Parietal Operculum Cortex, R	2	3.37	48	−24	30
Postcentral Gyrus, R	2	3.35	56	−18	40
*Cluster 1: 1029 voxels*					
Precuneous Cortex, R	1	3.25	14	−36	44
Precuneous Cortex, L	1	3.20	−10	−52	66
Posterior Cingulate Gyrus, R	1	3.10	6	−36	46
Superior Parietal Lobule, R	1	3.01	14	−50	64
Lateral Occipital Cortex (Sup), R	1	2.91	24	−58	52

^a^Presents the brain areas where the brain activation to correct inhibition to lures was significantly greater in the Amnestic/Non-Amnestic compared to Amnestic type groups on fMRI. ^b^Presents the brain areas where the brain activation to correct inhibition to lures was significantly greater in the Amnestic/Non-Amnestic compared to the Unimpaired type group on fMRI. A ‘Cluster’ is a group of anatomically adjacent voxels found to be statistically significant after thresholding with z-threshold = 2.3 and p = 0.05. Z-score represents the magnitude of group differences in activation and Montreal Neurological Institute (MNI) coordinates (x, y and z axis) represent a standardized way of presenting the anatomical location of the peak Z-score on a normalized brain template.

**Table 4:** Group differences in brain metabolite creatine ratios in the anterior cingulate cortex.

Metabolite creatine ratios	Based on underlying disease		Based on neuropsychological division		
	Non-cirrhotic	Cirrhosis	Unimpaired	Amnestic type	Amnestic+ Non-amnestic type
MyoinositoI	0.794 ± 0.08	0.593 ± 0.19‡‡	0.727 ± 0.15	0.824 ± 0.10	0.611 ± 0.19†
N-Acetyl Aspartate	1.303 ± 0.07	1.222 ± 0.12‡	1.294 ± 0.11*	1.266 ± 0.09	1.173 ± 0.09††
Glutamate+ Glutamine	1.812 ± 0.25	2.105 ± 0.47‡	1.955 ± 0.30	1.796 ± 0.29	1.897 ± 0.57

^‡^p < 0.05, ^‡‡^p < 0.01 between cirrhotic and non-cirrhotic, ^*^p < 0.05, **p < 0.01. Unimpaired vs. amnestic + non-amnestic type; ^†^p < 0.05, ^††^p < 0.01 amnestic vs. amnestic + non−amnestic type.

**Table 5:** Group differences on brain volumetric analysis.

Brain volume region	Based on the disease		Brain volume region	Based on neuro-psychological performance		
	Non-cirrhotic	Cirrhosis		Unimpaired	Amnestic type	Amnestic+ Non-amnestic
White matter	6.87 ± 0.4	6.68 ± 0.5	White matter	6.99 ± 0.2**	6.34 ± 0.6†	6.88 ± 0.2
Gray matter	6.41 ± 0.5	6.34 ± 0.7	Gray matter	6.59 ± 0.5*	5.99 ± 0.6†	6.55 ± 0.4
Total brain	13.3 ± 0.7	13.0 ± 1.2	Total brain	13.6 ± 0.6**	12.4 ± 1.25†	13.4 ± 0.8
			Hippocampus L	3.63 ± 0.4**	3.14 ± 0.5††	3.68 ± 0.3
			Hippocampus R	3.80 ± 0.5	3.50 ± 0.4†	3.86 ± 0.3
			Thalamus L	6.75 ± 0.5*	6.09 ± 0.7††	7.05 ± 0.6
			Thalamus R	6.53 ± 0.5	6.18 ± 0.4††	6.81 ± 0.5

^*^p < 0.05, **p < 0.01. Unimpaired vs. amnestic type; ^†^p < 0.05, ^††^p < 0.01 amnestic vs. amnestic/non-amnestic type. For total brain volumes (white matter, gray matter and total brain) data are presented as x 10^−5^ mm^3^ while for local volumes (Hippocampus and Thalamus) data are presented as x 10^−3^ mm^3^. All subjects’ volumes are reported relative to a normalized skull size.
