# Supplementary material for: Elderly patients have an altered gut-brain axis regardless of the presence of cirrhosis
Source: Sci Rep. 2016 Dec 6;6:38481. doi: 10.1038/srep38481 (PMC5138827; doi:10.1038/srep38481)
Supplement: Supplementary Information [file srep38481-s1.pdf]

# Elderly patients have an altered gut-brain axis regardless of the presence of cirrhosis

Jasmohan S Bajaj, MD[1*], Vishwadeep Ahluwalia, PhD[1], Joel L. Steinberg, MD[2], Sarah Hobgood, MD[3], Peter A Boling, MD[3], Michael Godschalk, MD[3], Saima Habib, MD[3], Melanie B White, RN[1], Andrew Fagan, BS[1], Edith A Gavis, RN[1], Dinesh Ganapathy, MD[1], Phillip B Hylemon, PhD[4], Karen E Stewart, PhD[2], Raffi Keradman[5], Eric J Liu[5], Jessica Wang[5], Patrick M Gillevet, PhD[5], Masoumeh Sikaroodi, PhD[5], F Gerard Moeller, MD[2], James B Wade, PhD[2]

[1]Division of Gastroenterology, Hepatology and Nutrition, [2]Department of Psychiatry, [3] Division of Geriatrics, [4]Department of Microbiology and Immunology, Virginia Commonwealth University and McGuire VA Medical Center, Richmond, VA, USA,

[5]Microbiome Analysis Center, George Mason University, Manassas, VA, USA

**Keywords:** Hepatic Encephalopathy, Microbiota, Dementia, Mild Cognitive Impairment, MRI

**Corresponding Author:**

Jasmohan S Bajaj, MD

Division of Gastroenterology, Hepatology and Nutrition,

Virginia Commonwealth University and McGuire VA Medical Center,

Richmond, Virginia, USA 23249

Telephone: (804) 675-5802

Fax: (804) 675-5816

Email: jsbajaj@vcu.edu

**Supplementary Materials**

1. **Brain Imaging Measures**

**MRI Sessions**. Prior to the MRI session, one practice run of ICT task was conducted outside the scanner. MRI Images were acquired using a Philips Ingenia 3 T MRI scanner with a 32-channel receive head coil (Philips Medical Systems, Best, Netherlands). Each MRI scanning session included the following scans: T1 weighted spin-echo 3-plane localizer (scout), high resolution T1 weighted 3D-MPRAGE scan (256 x 256 acquisition matrix, in-plane resolution 1 mm x 1 mm, 160 sagittal slices, 1 mm slice thickness (no gap), flip angle 6.0 degrees, minimum inversion delay 1133.3 ms; TR 8.55 ms, TE 4.0 ms, total duration 5 min 56 s) and 6 runs of the ICT task (described in detail below). Single voxel MR-Spectroscopy was performed over the Anterior Cingulate Cortex. A 3D-FLAIR scan was acquired during the MRI session of each subject for diagnostic purposes to be read by the radiologist to rule out incidental pathology.

**fMRI images for each ICT run** were acquired in the transverse plane using a single shot gradient-echo echo planar imaging (EPI) pulse sequence, repetition time = 2000 ms, echo time = 30 ms, flip angle 71 degrees, field-of-view = 240 mm × 240 mm, acquisition matrix = 64 x 64, in-plane resolution = 3.75 mm × 3.75 mm, number of axial slices = 35, slice thickness = 3.75 mm, no interslice gap, SENSE factor = 2, number of repetitions = 83 after 5 discarded (dummy) acquisitions, total scan duration per run (fMRI series) = 2 min 56 s. There was approximately 1 min of rest between each ICT run.

**ICT FMRI** is a rapid presentation event-related fMRI design that tests the ability of a subject to correctly respond to targets and withhold responses to lures. During the ICT, a stream of letters were rear-projected serially onto a MR compatible screen via a computer running Presentation software (NeuroBehavioral Systems, California). Subjects viewed the screen via a mirror placed over the head coil and responded using a button located near their right hand. Each letter was presented for 500 ms, with no interstimulus interval. Letters were classified as targets (x and y) to which the subject responds, lures (x and y), to which the subject inhibits a response, and

distractors (all other letters), which the subject ignores.  Subjects were instructed to respond to alternating x's and y's; i.e. respond if the current x is preceded by a y, or if the current y is preceded by an x.  Subjects were instructed to inhibit their response if the pattern does not alternate; i.e. inhibit if the current x is preceded by an x, or if the current y is preceded by a y.  On average, targets were presented every 3.5 sec and lures every 20 sec.  A minimum of 15 sec separates consecutive lures.  For the entire experiment, there were 212 targets, 40 lures, and 1248 distractors presented.  Response prepotency was maintained by including many more targets than lures and by instructing subjects to respond quickly. The ICT was well suited to the scanner environment in which subjects view a simple visual stimulus and respond with a single button press.

**1H-MR Spectra** were acquired using Point Resolved Spectroscopy (PRESS) sequence with a repetition time=1500ms, echo time=30ms and number of averages=128. Spectroscopic volume in the anterior cingulate cortex (20x20x20 $mm^3$ ) was prescribed on high-resolution structural images from 3D-MPRAGE in 3 planes. A Chemical-Shift Selective or CHESS sequence was used within PRESS to achieve water signal suppression. A separate PRESS sequence was acquired without water suppression, with same parameters except with only 8 averages. This was done to have a water peak reference for correction during spectral analysis.

**fMRI Processing and analysis of ICT fMRI:** Each of the ICT fMRI runs was visually inspected for quality control. fMRI data analysis for both tasks was carried out using FEAT (FMRI Expert Analysis Tool) v 5.98 part of FSL (FMRIB's Software Library, www.fmrib.ox.ac.uk/fsl) [1-3]._The following pre-statistics processing was applied; motion correction using MCFLIRT, non-brain removal using BET spatial smoothing using a Gaussian kernel of FWHM 6.0mm; grand-mean intensity normalization of the entire 4D dataset by a single multiplicative factor; highpass temporal filtering (Gaussian-weighted least-squares straight line fitting, with sigma=50.0s. Subjects that have >1.5mm absolute motion were removed from further analysis. After preprocessing, a time-series statistical analysis was carried out using FILM with local autocorrelation correction. First level time series statistical analysis was carried out on each of the six ICT runs separately. Stimulus timings for Correct Response to Target (CRT), Correct

Inhibition to Lures (CIL), Incorrect Response to Lures (IncRL) and random responses (RANDOM) were extracted from each individual response files recorded by the Presentation software. Regressors were created by convolving these events by a gamma hemodynamic response function. A general linear model was specified that includes CRT, CIL, IncRL and RANDOM as regressors of interest and motion parameters as confound regressors. Contrast maps were created for each condition vs. Baseline (Fixation + Non-Target letters) and registered to high-resolution structural and the 152 brain average Montreal Neurological Institute (MNI) standard space template using linear (FLIRT) and nonlinear (FNIRT) registration methods[1, 4]. Contrast images for CIL, CRT and IncRL were passed on to the higher-level analysis. Higher-level analysis was performed in two steps. The first step combines the 6 runs at the subject level using a standard weighted fixed effects model to form a single statistic image for the CIL condition per subject. These within-subject 'averaged' parameter estimates were fed to higher-level analysis comparing activation to correct inhibition, between groups. This was done using FLAME (FMRIB's Local Analysis of Mixed Effects) stage 1[5, 6] and group difference z-score brain maps were generated and thresholded using a cluster-based threshold[7].

**Analysis of 1H-MR Spectroscopy using LCModel:** Only those spectra with autoshim linewidth of < 10Hz were acquired and considered for analysis. Among other metabolites, Creatine (Cr), myo-inositol (mI), glutamate+glutamine (Glx), Choline (Cho) and N-acetylaspartate (NAA) complex peak areas were computed along with their respective Cramer-Rao lower bounds, using a quantitative assessment of the metabolite concentration by means of LCModel software [8, 9]. Concentration ratios were computed with respect to Creatine. Ratios to an internal reference such as Cr greatly reduces the effect of arbitrary inter-scan signal variations. Only those metabolites ratios with corresponding Cramer'-Rao lower bound < 20% were considered for further analysis as a measure of quality control except for Glutamine whose %SD threshold was relaxed to 25% since it is less readily detected. LCModel utilizes a basis set of reference *in vitro* MR spectra for all major metabolites to deduce absolute concentrations of corresponding compounds from *in vivo* MR brain spectra. The model corrects for residual eddy current and RF coil loading effects and allows for an estimate and subtraction of the spectral baseline

nonlinearity which is normally present at the short echo time (TE) used in this study. Details of the method and reproducibility of the technique have been described by Provencher et al. (10-11). Group comparisons were done using standard t-tests in SPSS.

**Volumetric analysis** Brain tissue volume, normalised for subject head size, was estimated with SIENAX part of FSL. SIENAX starts by extracting brain and skull images from the single whole-head input data. The brain image is then affine-registered to MNI152 space (using the skull image to determine the registration scaling); this is primarily in order to obtain the volumetric scaling factor, to be used as a normalisation for head size. Next, tissue-type segmentation with partial volume estimation is carried out in order to calculate total volume of brain tissue (including separate estimates of volumes of gray matter, white matter, peripheral gray matter and ventricular CSF). Additionally, we also estimated Hippocampal and Thalamic volumes using FIRST (part of FSL) and normalised them for head size. FIRST is a model-based segmentation/registration tool. The shape/appearance models used in FIRST are constructed from manually segmented images provided by the Center for Morphometric Analysis (CMA), MGH, Boston. Based on their learned models, FIRST searches through linear combinations of shape modes of variation for the most probable shape instance given the observed intensities in a T1-weighted image.

## 2. Microbiota analysis:

Microbiota: Stool was collected and DNA extracted using published techniques[10]. We first used Length Heterogeneity PCR (LH-PCR) fingerprinting of the 16S rRNA to rapidly survey our samples and standardize the community amplification. We then interrogated the microbial taxa associated using Multitag Pyrosequencing (MTPS) [11]. This technique allows the rapid sequencing of multiple samples at one time. Microbiome Community Fingerprinting: About 10 ng of extracted DNA was amplified by PCR using a fluorescently labeled forward primer 27F (5'-(6FAM) AGAGTTTGATCCTGGCTCA G-3') and unlabeled reverse primer 355R' (5'-

GCTGCCTCCCGTAGGAGT-3'). Both primers are universal primers for bacteria. The LH-PCR products were diluted according to their intensity on agarose gel electrophoresis and mixed with ILS-600 size standards (Promega) and HiDi Formamide (Applied Biosystems, Foster City, CA). The diluted samples were then separated on a ABI 3130xl fluorescent capillary sequencer (Applied Biosystems, Foster City, CA) and processed using the Genemapper™ software package (Applied Biosystems, Foster City, CA). Normalized peak areas were calculated using a custom PERL script and operational taxonomic units (OTUs) constituting less than 1% of the total community from each sample were eliminated from the analysis to remove the variable low abundance components within the communities.

MTS[11]: Specifically, we have generated a set of 96 emulsion PCR fusion primers that contain the 454 emulsion PCR linkers on the 27F and 355R primers and a different 8 base "barcode" between the A adapter and 27F primer. Thus, each fecal sample was amplified with unique bar-coded forward 16S rRNA primers and then up to 96 samples were pooled and subjected to emulsion PCR and pyrosequenced using a GS-FLX pyrosequencer (Roche). Data from each pooled sample were "deconvoluted" by sorting the sequences into bins based on the barcodes using custom PERL scripts. Thus, we were able to normalize each sample by the total number of reads from each barcode. We have noted that ligating tagged primers to PCR amplicons distorts the abundances of the communities and thus it is critical to incorporate the tags during the original amplification step. Microbiome Community Analysis: We identified the taxa present in each sample using the Bayesian analysis tool in Version 10 of the Ribosomal Database Project (RDP10). The abundances of the bacterial identifications were then normalized using a custom PERL script and genera present at >1% of the community were tabulated. We chose this cutoff because of our *a priori* assumption that genera present in < 1% of the community vary between individuals and have minimal contribution to the functionality of that community and 2,000 reads per sample will only reliably identify community components that are greater than 1% in abundance.

Analysis of microbiota: LEFSe was used to evaluate changes in overall microbial abundance [12].

**3. Supplementary Table: Details of cirrhosis sub-group**

| Variable (mean ± SD unless otherwise mentioned) | N=39 |
|---|---|
| Age (years) | 72.9±5.4 |
| MELD score | 8±4 |
| Child-Turcotte-Pugh Score | 6±2 |
| Etiology (Hepatitis C/Alcohol/NAFLD/Others) | 13/11/14/1 |
| Ascites (none/controlled on diuretics/uncontrolled) | 35/4/0 |
| Prior variceal bleeding | 5 |
| Beta-blocker use | 8 |
| Score <-4 on PHES per local norms | 18 |

## Supplementary references


1. Jenkinson M, Bannister P, Brady M, Smith S. Improved optimization for the robust and accurate linear registration and motion correction of brain images. NeuroImage 2002;17:825-841.
2. Smith SM. Fast robust automated brain extraction. Human brain mapping 2002;17:143-155.
3. Woolrich MW, Ripley BD, Brady M, Smith SM. Temporal autocorrelation in univariate linear modeling of FMRI data. NeuroImage 2001;14:1370-1386.
4. Jenkinson M, Smith S. A global optimisation method for robust affine registration of brain images. Medical image analysis 2001;5:143-156.
5. Beckmann CF, Jenkinson M, Smith SM. General multilevel linear modeling for group analysis in FMRI. NeuroImage 2003;20:1052-1063.
6. Woolrich M. Robust group analysis using outlier inference. NeuroImage 2008;41:286-301.
7. Woolrich MW, Ripley BD, Brady M, Smith SM. Temporal autocorrelation in univariate linear modeling of FMRI data. Neuroimage 2001;14:1370-1386.
8. Provencher SW. Estimation of metabolite concentrations from localized in vivo proton NMR spectra. Magn Reson Med 1993;30:672-679.
9. Provencher SW. Automatic quantitation of localized in vivo 1H spectra with LCModel. NMR Biomed 2001;14:260-264.
10. Bajaj JS, Hylemon PB, Ridlon JM, et al. Colonic mucosal microbiome differs from stool microbiome in cirrhosis and hepatic encephalopathy and is linked to cognition and inflammation. Am J Physiol Gastrointest Liver Physiol 2012;303:G675-685.
11. Gillevet P, Sikaroodi M, Keshavarzian A, Mutlu EA. Quantitative assessment of the human gut microbiome using multitag pyrosequencing. Chem Biodivers 2010;7:1065-1075.
12. Segata N, Izard J, Waldron L, et al. Metagenomic biomarker discovery and explanation. Genome Biol 2011;12:R60.
